# The Impact of Pre- Versus Post-Transplant Multidrug-Resistant Organism Colonization on Liver Transplant Outcomes: A Retrospective Cohort Study

**DOI:** 10.3390/diseases14100362

**Published:** 2026-10-01

**Authors:** Emil Aliev, Efrat Orenbuch-Harroch, Abed Khalaileh, Ashraf Imam, Matan Joel Cohen, Maya Korem

**Affiliations:** 1 Institute for Research in Military Medicine, Department of Military Medicine, Faculty of Medicine, The Hebrew University of Jerusalem, Jerusalem 91120, Israel; emil.aliev@mail.huji.ac.il; 2 The Israel Defense Forces Medical Corps, Jerusalem 5262000, Israel; 3 Department of Clinical Microbiology and Infectious Diseases, Hadassah Medical Center, Jerusalem 91120, Israel; orenef@gmail.com; 4 Faculty of Medicine, Hebrew University of Jerusalem, Jerusalem 91120, Israel; hbedk@hadassah.org.il (A.K.); achrafim@hadassah.org.il (A.I.); matanc123@gmail.com (M.J.C.); 5 Department of Medical Intensive Care, Hadassah Medical Center, Jerusalem 91120, Israel; 6 Transplantation Unit, Hadassah Medical Center, Jerusalem 91120, Israel; 7 Clalit Health Services, Jerusalem District, P.O. Box 27670, Jerusalem 9127601, Israel

**Keywords:** liver transplantation, multidrug-resistant organisms, post-transplant infection, colonization, one-year mortality

## Abstract

Objectives: Infectious complications, particularly those caused by multidrug-resistant organisms (MDROs), remain a major cause of morbidity and mortality after liver transplantation (LT). While pre-transplant colonization is a recognized risk factor, its prognostic significance relative to post-transplant MDRO status remains unclear. This study evaluated the clinical impact of MDRO presence across the transplant timeline and identified predictors of post-transplant mortality and graft dysfunction. Methods: A retrospective cohort study of 110 adult LT recipients (2018–2024) was conducted at a tertiary center. Patients were stratified by pre-transplant ESBL/MDRO colonization or infection and by post-transplant MDRO status (modeled as a time-dependent exposure). Primary outcomes were one-year all-cause mortality and adverse graft outcomes. Results: Pre-transplant ESBL/MDRO presence (40% of recipients) was not associated with increased one-year mortality or adverse graft outcomes. Conversely, post-transplant MDRO status occurred in 44.5% of patients and was strongly associated with poor outcomes. Post-transplant MDRO colonization or infection was present in 88% (14/16) of non-survivors vs. 37% (35/94) of survivors (*p* < 0.001). One-year mortality was 47% (9/19) for post-transplant MDRO infection, 17% (5/30) for colonization alone, and 3% (2/61) with no post-transplant MDRO event. In a penalized time-dependent Cox model, post-transplant MDRO acquisition was independently associated with one-year mortality (HR 4.0, 95% CI 1.77–9.03; *p* < 0.001), confirmed by landmark analysis (HR 3.15, 95% CI 1.08–9.19), whereas pre-transplant status showed no association (*p* = 0.18). Conclusions: While pre-transplant status did not significantly impact one-year mortality in this cohort, post-transplant MDRO acquisition was independently associated with one-year mortality. Pre-transplant status should guide targeted prophylaxis, whereas the post-transplant period represents the critical window for infection prevention and antimicrobial stewardship interventions.

## 1. Introduction

Liver transplantation (LT) is a life-saving procedure for patients with end-stage liver disease and acute liver failure, significantly improving survival and quality of life [1,2]. However, post-transplant infections, particularly those caused by multidrug-resistant organisms (MDROs), remain a major threat to both patient and graft outcomes. Infectious complications are the leading cause of morbidity in the early post-LT period, with bacterial and fungal infections accounting for nearly two-thirds of sepsis-related deaths and 37–59% of mortality within 30–180 days after transplant [3,4,5,6,7,8].

LT recipients are exposed to numerous risk factors for MDRO acquisition and infection across the pre-, intra-, and post-transplant period. Pre-transplant risk factors include prolonged hospitalization, intensive care unit (ICU) admission, broad-spectrum antibiotic exposure, indwelling devices, renal replacement therapy, and notably pre-existing MDRO colonization [5,6,7,9].

Post-transplant risks include prolonged mechanical ventilation, extended ICU stay, renal replacement therapy, biliary complications, surgical reintervention, acute rejection requiring intensified immunosuppression, cytomegalovirus (CMV) infection, delayed graft function, and early re-transplantation [5,6,7,9].

The clinical consequences of MDRO infections are substantial. Reported mortality varies by pathogen, ranging from 15 to 28% for ESBL-producing *Enterebacrerales* to 30–70% for carbapenem-resistant *Enterobacterales*, 40–65% for multidrug-resistant *Pseudomonas aeruginosa* and *Acinetobacter baumannii*, and 6–60% for methicillin-resistant *Staphylococcus aureus* [5,7,8]. Beyond mortality, MDRO infections are associated with increased graft dysfunction, prolonged ICU and hospital stays, higher healthcare costs, and greater need for surgical or immunosuppressive interventions. A large European cohort demonstrated significantly lower infection resolution rates, higher rates of severe sepsis or shock, increased acute-on-chronic liver failure, and markedly higher 28-day mortality among patients with MDRO infections compared to non-MDRO infections [5,9,10,11].

The objective of this study was to characterize the clinical impact of MDRO colonization and infection in LT recipients across the pre- and post-transplant periods and to identify risk groups associated with adverse outcomes following transplantation.

## 2. Materials and Methods

### 2.1. Study Design and Patient Population

This retrospective cohort study included adult patients (≥18 years) who underwent LT at the Hadassah-Hebrew University Medical Center in Jerusalem, a 1300-bed tertiary hospital, between 1 April 2018 and 30 June 2024. The Solid Organ Transplantation Unit functions as one of Israel’s three national liver transplant centers, performing approximately 25 liver transplantations per year. Demographic, clinical, and microbiological data were extracted from the hospital’s electronic medical records system using a predefined, structured data collection form (Table 1). Mortality data were obtained from the national population registry and used to assess survival outcomes.

### 2.2. Microbiology

Screening for MDRO colonization was performed according to hospital protocol using routine rectal swab cultures obtained on admission to the transplant unit and at the discretion of the treating team during subsequent admissions. Rectal swabs were inoculated onto chromogenic selective media for MDRO detection, with organism identification by Matrix-Assisted Laser Desorption/Ionization Time-of-Flight Mass Spectrometry (MALDI-TOF MS, bioMerieux, Marcy, I’Etoile, France) and carbapenemase gene detection by multiplex PCR when indicated. Antibiotic susceptibility testing was conducted according to Clinical Laboratory Standards Institute (CLSI) guidelines using an automated method [12].

### 2.3. Definitions

Multidrug-resistant organisms (MDROs) were defined operationally, in line with standardized criteria for acquired multidrug resistance [12,13], as non-susceptibility to at least one agent in a major antibiotic class, confirmed by phenotypic susceptibility testing performed according to CLSI guidelines [12,13]. The following organism groups were included, each meeting the following criteria: (i) methicillin-resistant *Staphylococcus aureus* (MRSA), (ii) vancomycin-resistant *Enterococcus* (VRE), (iii) carbapenem-resistant *Enterobacterales* (CRE), defined as non-susceptibility to at least one carbapenem without identification of a carbapenemase, and (iv) carbapenemase-producing *Enterobacterales* (CPE), defined by detection of a carbapenemase gene via polymerase chain reaction (PCR). The specific criteria met by each organism group are summarized in Supplementary Table S1.

An isolate was considered phenotypically consistent with extended-spectrum β-lactamase (ESBL) production when it demonstrated reduced susceptibility or resistance to third-generation cephalosporins.

Colonization was defined as the isolation of an MDRO from screening or surveillance cultures (e.g., rectal swabs) in the absence of clinical signs or symptoms of infection. Infection was defined according to treating-physician documentation supported by compatible clinical, laboratory, and radiologic findings.

Pre-transplant MDRO status referred to any documented colonization or infection occurring within 12 months prior to transplantation. Pre-transplant ESBL infection was defined as any documented clinical infection caused by an ESBL-producing *Enterobacterales* isolate within 12 months prior to transplantation. For analysis, pre-transplant MDRO status and pre-transplant ESBL infection were grouped as pre-transplant ESBL/MDRO colonization or infection.

Post-transplant MDRO status included any new colonization or infection identified during the index hospitalization or within 12 months following transplantation. For the primary survival analysis, it was modeled as a time-dependent exposure and post-transplant events were additionally classified as MDRO infection or MDRO colonization alone and analyzed separately (Supplementary Figure S1). Post-transplant ESBL infections and their sites were analyzed descriptively. Infection sites were categorized as bloodstream, intra-abdominal/biliary, urinary/pulmonary surgical site, or other (Supplementary Table S2).

Adverse graft outcomes were defined as primary failure, rejection, or re-transplantation during the index hospitalization or within 12 months following transplantation.

### 2.4. Statistical Analysis

Statistical analyses were performed using IBM SPSS Statistics (version 31.0). Patients were stratified and compared based on two primary groups: pre-transplant ESBL/MDRO colonization or infection and post-transplant MDRO status. Group comparisons were conducted using the chi-square test or Fisher’s exact test, as appropriate, for categorical variables and the *t*-test/Mann–Whitney test for quantitative continuous variables. Effect sizes are reported alongside *p*-values: categorical comparisons as absolute risk differences with 95% confidence intervals and continuous comparisons as mean differences. Survival curves were generated using the Kaplan–Meier method and compared with the log-rank test. Because post-transplant MDRO status was defined over the same follow-up period in which mortality occurred, it was modeled as a time-dependent covariate in a Cox proportional hazards framework: each patient contributed unexposed person-time from the date of transplantation until the first post-transplant MDRO culture (colonization or infection) and exposed person-time thereafter. Follow-up was censored at 365 days. Because 14 of the 16 deaths occurred after MDRO acquisition, the unpenalized model exhibited complete separation; a penalized (Firth-type) Cox model was therefore fitted, both univariably and adjusted for age and Model for End-Stage Liver Disease (MELD) score. The model was deliberately restricted to three covariates, consistent with the events-per-variable constraint imposed by 16 deaths. Proportional hazards were assessed using Schoenfeld residuals; the time-dependent specification of the MDRO exposure was adopted specifically to accommodate the non-proportionality implied by a fixed 12-month exposure window. Two additional analyses addressed time-related bias. First, a 30-day landmark analysis included patients alive at day 30 after transplantation, with exposure defined by MDRO cultures obtained before day 30. Second, a sensitivity analysis restricted the post-transplant MDRO exposure window to 3 months, consistent with the prior literature [13]. Finally, post-transplant MDRO infection and MDRO colonization alone were analyzed as separate exposures. All statistical tests were two-tailed, and a *p*-value ≤ 0.05 was considered statistically significant.

## 3. Results

### 3.1. Cohort Characteristics

During the study period, 110 patients underwent LT. The mean age was 54.1 ± 14.8 years and 69 (63%) were male. The leading indication for transplantation was non-alcoholic fatty liver disease (NAFLD) (41.8%), followed by viral hepatitis (19%) and autoimmune hepatitis (12.7%). At the time of transplantation, 44 (40%) had documented pre-transplant ESBL infection, MDRO colonization or MDRO infection (Table 1).

Patients with pre-transplant ESBL/MDRO colonization or infection had significantly higher MELD scores and greater preoperative healthcare exposure than those without colonization or infection. This included more prior hospitalizations and longer cumulative pre-transplant hospital stays. They also received more antibiotics in the three months preceding transplantation. During transplantation, piperacillin–tazobactam or carbapenem prophylaxis was administered more frequently and for longer durations. These patients required more peri-transplant blood transfusions, had longer ICU stays, and experienced higher rates of septic shock and post-transplant infectious complications, including bloodstream infections (BSIs) and ESBL or MDRO infections. Within the first year after transplantation, 33.3% (22/66) of patients without pre-transplant ESBL/MDRO colonization or infection became colonized with MDRO, and 4.5% (3/66) developed MDRO infection, compared to 61% (27/44) and 36% (16/44), respectively, among those with pre-transplant ESBL/MDRO colonization or infection (*p* < 0.001) (Table 1). These findings demonstrate a strong association between pre-transplant colonization with resistant bacteria and post-transplant MDRO status, with recipients colonized or infected before transplantation being substantially more likely to develop subsequent MDRO colonization or infection after transplantation.

**Figure 1:**
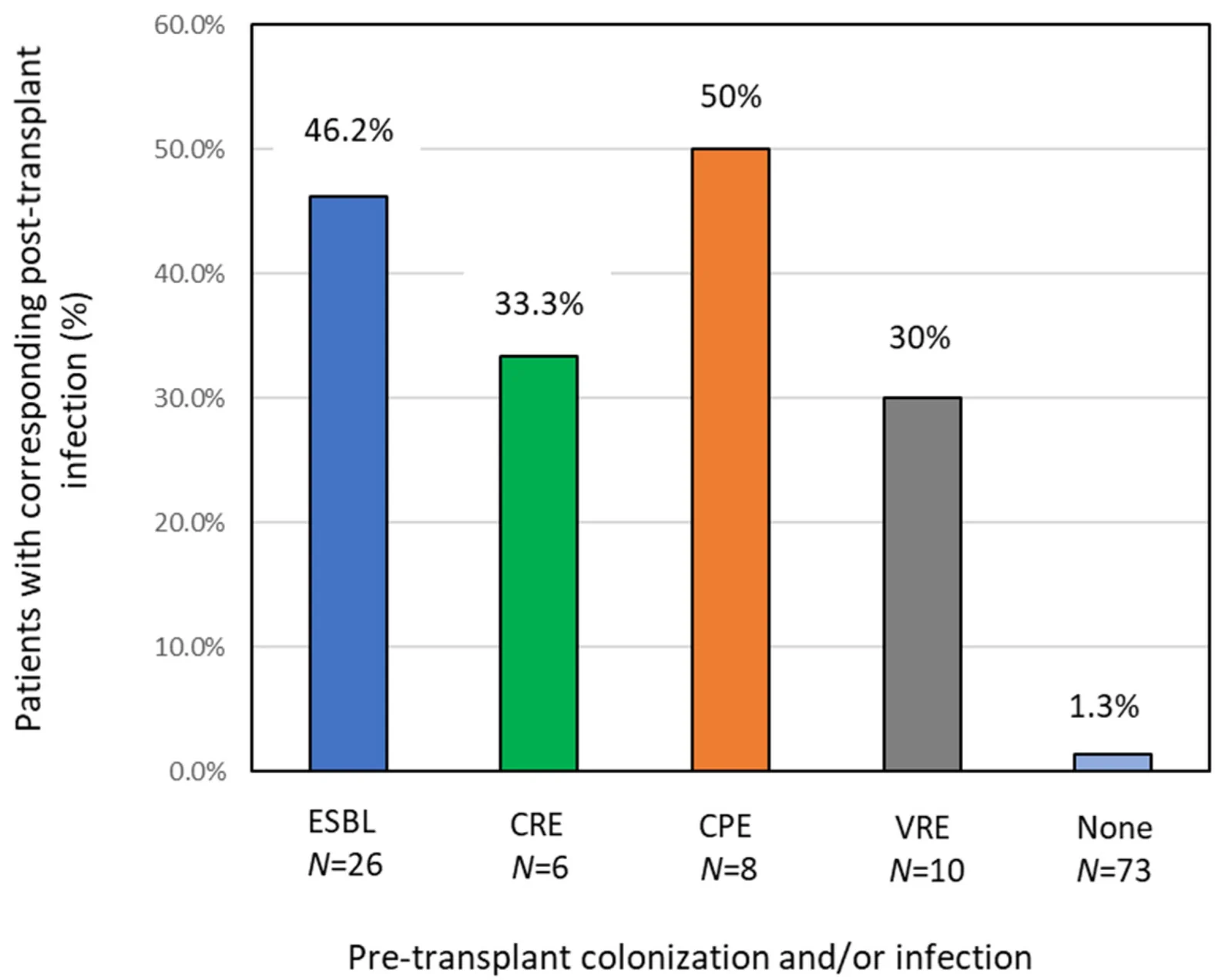
Concordance between pre-transplant ESBL/MDRO colonization or infection and corresponding post-transplant resistant infection.

**Figure 2:**
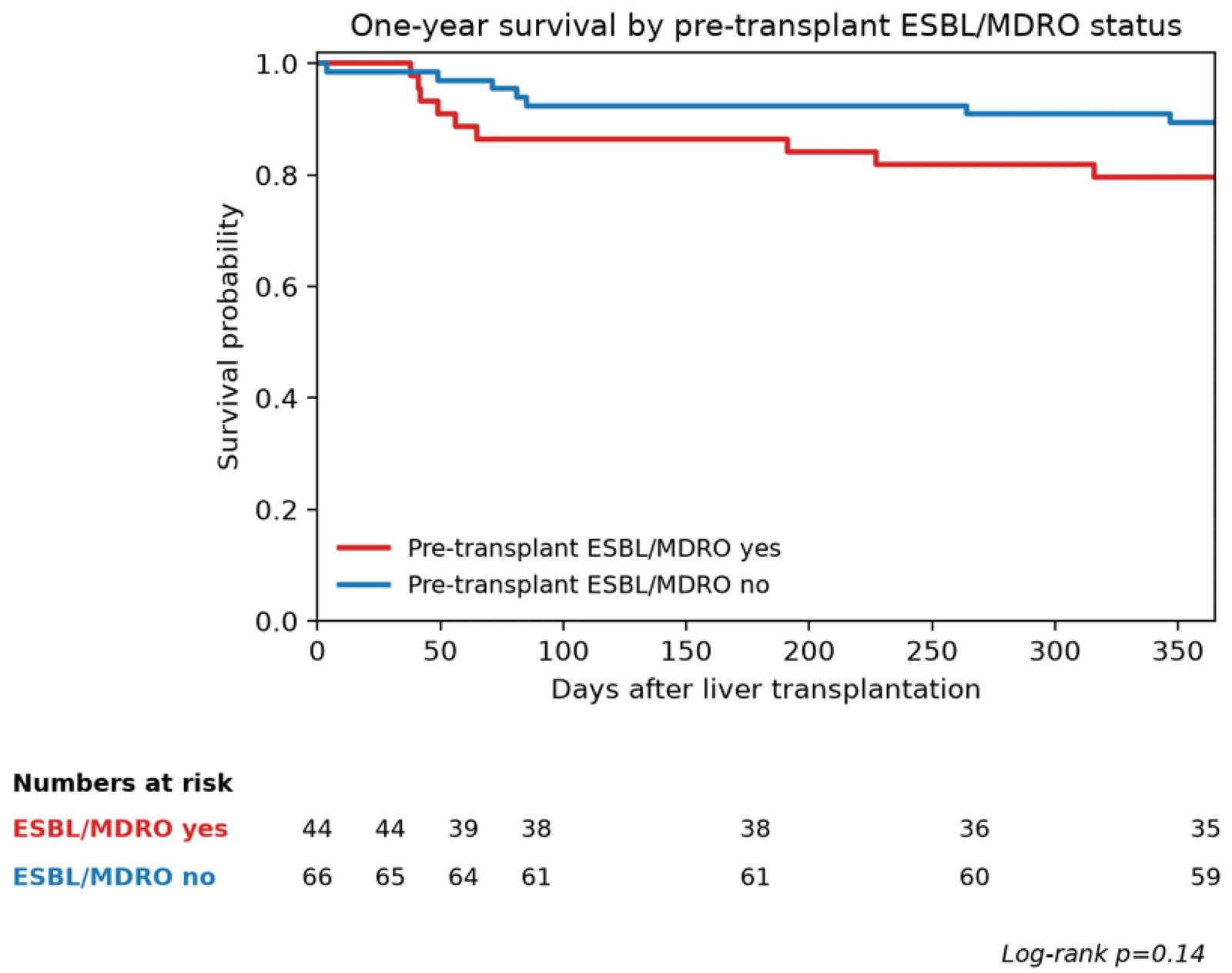
One-year post-transplant survival by pre-transplant ESBL/MDRO colonization or infection. Probability of survival during the first year after transplantation in patients with and without pre-transplantation ESBL/MDRO colonization or infection (*p* = 0.14).

To evaluate the microbiological relationship between pre-transplant ESBL/MDRO colonization or infection and subsequent post-transplant infection, organism-specific analyses were performed (Figure 1): Among patients with pre-transplant ESBL infection, 46.2% (12/26) subsequently developed post-transplant ESBL infection. Similarly, corresponding post-transplant infections occurred in 33.3% (2/6) of patients with pre-transplant CRE colonization/infection, 50.0% (4/8) of patients with pre-transplant CPE colonization/infection, and 30.0% (3/10) of patients with pre-transplant VRE colonization/infection. In contrast, corresponding resistant infections were uncommon among patients without prior colonization or infection. A significant overall association was observed between pre-transplant resistant organism carriage and subsequent infection with the same resistance phenotype (Fisher’s exact test, *p* < 0.0001). There was no statistically significant association between pre-transplant ESBL/MDRO colonization or infection and one-year all-cause mortality (Figure 2, Table 1). Among the 16 patients who died within the first year after LT, 21% (*n* = 9) had pre-transplant ESBL/MDRO colonization or infection, as did 37% (*n* = 35) of survivors (*p* = 0.18). Pre-transplant status was not significantly associated with graft outcomes: 50% (19/38) of patients with adverse graft outcomes had ESBL/MDRO colonization or infection, compared with 34% (25/72) among those with successful graft outcomes (*p* = 0.15).

Post-transplant MDRO status was observed in 44.5% (49/110) of the cohort and in 30.2% (23/76) of patients who were not colonized with an MDRO at the time of LT. Compared with patients without post-transplant MDRO, these individuals had significantly longer ICU stays, more ICU-related complications, including prolonged mechanical ventilation and hemodialysis, greater pre-transplant exposure to intravenous antibiotics, and more frequent use of carbapenem prophylaxis.

Patients with post-transplant MDRO status experienced a markedly higher burden of infectious complications. They had significantly more BSIs, intra-abdominal or biliary infections, and septic shock during the transplant admission, as well as higher rates of ESBL/MDRO infections and fungal infections during the first post-transplant year.

**Figure 3:**
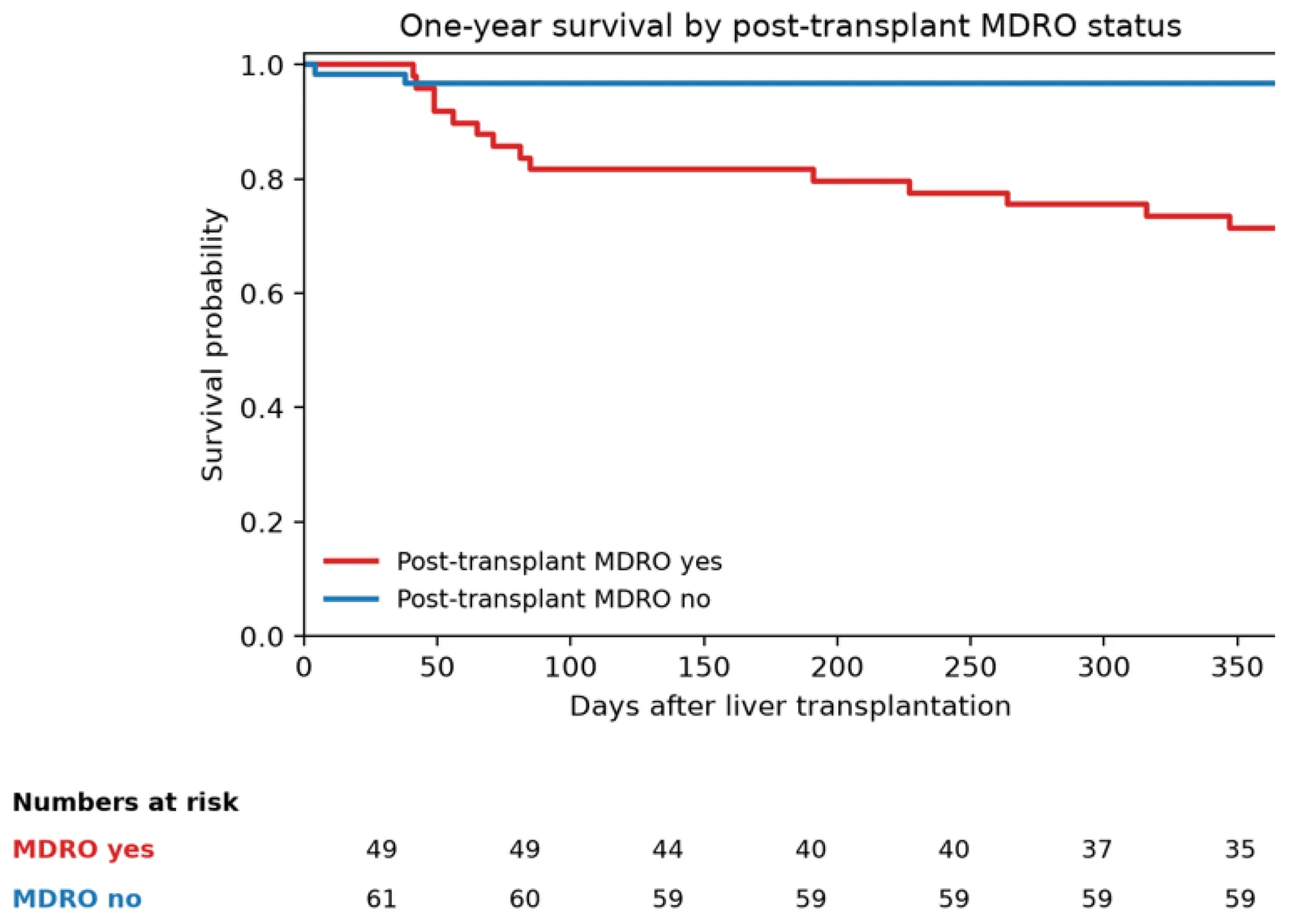
One-year post-transplant survival by post-transplant MDRO status (colonization and/or infection) (log-rank *p* ≤ 0.001).

Unlike pre-transplant status, post-transplant MDRO status was strongly associated with one-year mortality: 87% (14/16) of patients who died within the first year had post-transplant MDRO colonization or infection, compared with 37% (35/94) of survivors (absolute difference 50 percentage points; 95% CI 26 to 75; *p* < 0.001) (Table 2, Figure 3). Because post-transplant MDRO status and mortality were ascertained over the same follow-up period, this association was re-examined in time-dependent and landmark models as described below.

### 3.2. Multivariate Analysis and Survival Predictors

(i) Infection sites and colonization versus infection

Among the 49 patients with a post-transplant MDRO status, 19 had an MDRO infection (CRE 4, CPE 9, VRE 6) and 30 had MDRO colonization alone. The sites of the 19 MDRO infections and of post-transplant ESBL infections are detailed in Supplementary Table S2. One-year mortality was 9/19 (47%) among patients with MDRO infection, 5/30 (17%) among patients with MDRO colonization alone, and 2/61 (3%) among patients without post-transplant MDRO status (log-rank: MDRO infection vs. none, *p* = 0.002; colonization alone vs. none, *p* = 0.19; Supplementary Figure S1). The association with mortality was therefore driven by invasive MDRO infection rather than by colonization alone, though the colonization-only subgroup was small and this comparison is underpowered.

(ii) Time-dependent and landmark survival analyses

In a penalized time-dependent Cox model, in which post-transplant MDRO exposure began on the day of the first positive culture, MDRO acquisition remained independently associated with one-year mortality (univariable hazard ratio [HR] 4.32, 95% CI 1.93–9.63, *p* < 0.001; adjusted for age and MELD, HR 4.0, 95% CI 1.77–9.03, *p* < 0.001) (Supplementary Table S4). In this model, age and MELD score were not independently associated with mortality (age HR 1.06 per year, 95% CI 0.99–1.12, *p* = 0.09; MELD HR 1.01, 95% CI 0.91–1.12, *p* = 0.85). In the 30-day landmark analysis (*n* = 105 patients alive at day 30), MDRO acquisition before day 30 remained associated with mortality (HR 3.15, 95% CI 1.08–9.19, *p* = 0.035). In the sensitivity analysis restricting the exposure window to 3 months after transplantation, 11/35 (31%) of patients with an MDRO event within 3 months died within one year, compared with 5/75 (7%) without such an event (*p* = 0.001).

Temporality was further supported by Supplementary Table S3: among patients who died within one year, MDRO/ESBL infections were generally detected early after transplantation (median approximately 16 days) and preceded death by several weeks in most cases, and 13/16 deaths were adjudicated as infection-related. Nevertheless, causality cannot be inferred from this observational analysis: post-transplant MDRO acquisition may be both a consequence of a complicated postoperative course (prolonged ventilation, hemodialysis, ICU stay) and a mediator of subsequent mortality, and with 16 deaths a formal mediation analysis was not feasible. The multivariate estimates should therefore be interpreted as describing a robust association, not a proven independent causal effect.

## 4. Discussion

In this single-center cohort of LT recipients, we found that pre-transplant ESBL/MDRO colonization or infection was common, affecting 40% of recipients, and strongly predicted subsequent post-transplant MDRO acquisition, including infection with the same resistance phenotype. However, it was not associated with one-year all-cause mortality or adverse graft outcomes in this cohort—a finding that must be interpreted in light of the small number of deaths (*n* = 16) and the conditional nature of a transplant cohort and which cannot exclude a clinically important association. In contrast, post-transplant MDRO status was strongly associated with severe infectious complications and with one-year mortality, an association that persisted when post-transplant MDRO exposure was modeled as a time-dependent covariate and in a 30-day landmark analysis. These findings suggest that while pre-transplant colonization primarily reflects underlying disease severity and healthcare exposure, post-transplant MDRO status marks a high-risk trajectory and represents a critical, potentially modifiable determinant of post-transplant prognosis.

Patients with liver diseases, particularly cirrhosis, demonstrate immune dysfunction characterized by chronic systemic inflammation driven by increased gut bacterial translocation and profound immune deficiency with acquired immune system paralysis. This immune dysregulation, combined with elevated MELD scores, acute-on-chronic liver failure, hepatorenal syndrome, and prior spontaneous bacterial peritonitis (SBP), substantially increases susceptibility to infection [5,6,14,15,16]. Intraoperative factors such as prolonged operative time, extended cold ischemia time, complex biliary reconstruction, massive transfusion, and combined organ transplantation further contribute to this vulnerability. Within this context, the role of MDRO colonization or infection before and after transplantation becomes particularly relevant [9,17,18,19].

Our pre-transplant findings should be read alongside the existing literature. Prior research has identified pre-transplant MDRO colonization as a major predictor of post-transplant infections, with colonized patients being 5.5 to 12 times more likely to develop invasive infections with the same organisms following transplantation [5,6,7,10], and predictive models such as those of Giannella et al. and the INCREMENT-SOT-CPE score have emphasized the mortality risk with these infections [7,20,21,22,23,24]. Several larger cohorts and recent comprehensive reviews, including the review by Ferrarese et al., which documents substantial geographic heterogeneity in MDRO prevalence and predominant strains and the frequent interchangeability of colonization and infection data across studies [5], have reported that pre-transplant MDRO carriage, particularly when close to transplantation, is associated with post-transplant infection and inferior outcomes. Our cohort differs from these in size (110 recipients, 16 deaths) and in the 12-month exposure window, both of which limit our ability to detect a mortality signal. Consistent with previous reports, pre-transplant ESBL/MDRO colonization or infection was strongly associated with subsequent post-transplant MDRO acquisition (Table 1), and this association was organism-specific (Figure 1). Despite a higher infectious burden and greater healthcare exposure, colonized patients did not experience higher one-year mortality or poorer graft outcomes. We suggest three explanations: the study is underpowered to detect a moderate effect; patients with the most severe pre-transplant MDRO burden may not have survived to transplantation (a selection phenomenon inherent to transplant cohorts); and documented pre-transplant colonization at our center prompts intensified surveillance and tailored empirical antimicrobial therapy based on the recipient’s resistance profile, which may mitigate the downstream risk. Pre-transplant ESBL/MDRO status is therefore best regarded as a risk-stratification marker identifying patients at increased risk of post-transplant MDRO acquisition, guiding personalized infection prevention and stewardship strategies, rather than as an established independent mortality predictor.

A further finding was the strong organism-specific relationship between pre-transplant colonization or infection and subsequent post-transplant infection with the same resistance phenotype. Nearly half of patients with pre-transplant ESBL infection developed post-transplant ESBL infection, and similar patterns were observed for CRE, CPE, and VRE (Figure 1). These findings are consistent with prior studies demonstrating that colonization serves as a reservoir for later invasive infection in transplant recipients [21,25,26]. Although pre-transplant colonization was not independently associated with mortality in our cohort, its strong association with subsequent resistant infections suggests that its clinical importance may be mediated through increased susceptibility to post-transplant MDRO acquisition and infection.

In contrast, post-transplant MDRO colonization or infection was associated with an ominous prognosis. Post-transplant MDRO status occurred in 44.5% of recipients within the first year, including 30.2% of patients not colonized at the time of transplantation, and these patients experienced significantly higher rates of bloodstream infections, intra-abdominal and biliary infections, and septic shock during the index admission, as well as ESBL/MDRO and fungal infections during follow-up. The mortality signal was driven by invasive infection rather than colonization alone: one-year mortality was 47% (9/19) among patients with post-transplant MDRO infection, 17% (5/30) among patients with MDRO colonization alone, and 3% (2/61) among patients without post-transplant MDRO status (Supplementary Figure S1). Because post-transplant MDRO status and mortality were ascertained over the same follow-up period, we addressed this time-related bias formally: in a penalized time-dependent Cox model, with exposure beginning at the first positive culture, MDRO acquisition remained independently associated with one-year mortality (adjusted HR 2.99, 95% CI 1.37–6.51), and the association persisted in a 30-day landmark analysis (HR 3.59, 95% CI 1.11–11.63) and in a sensitivity analysis restricted to a 3-month exposure window. Among patients who died, MDRO/ESBL infections were typically identified early after transplantation (median approximately 16 days) and preceded death by several weeks in most cases, and 13 of 16 deaths were adjudicated as infection-related.

Whether post-transplant MDRO acquisition is an independent risk factor for death or a marker of a complicated postoperative course remains a central interpretive question, and our data speak to both possibilities. On the one hand, the association persisted after adjustment for age and MELD score, and temporality was supported by the sequence of events among decedents. On the other hand, post-transplant MDRO acquisition was strongly associated with prolonged ICU stay, mechanical ventilation, hemodialysis and septic shock, and it is biologically plausible that it lies on the causal pathway between surgical and critical-care morbidity and death; that is, the same interventions and complications that prolong critical illness also select for MDRO acquisition, which then contributes to mortality. With 16 deaths, a formal mediation analysis distinguishing these pathways was not feasible. We therefore present post-transplant MDRO status as a robust marker of a high-risk post-transplant trajectory and a potentially actionable target for intervention, rather than as a proven independent causal factor.

Our findings align with and extend current stakeholder guidance. The American Society of Transplantation Infectious Diseases Community of Practice guideline on multidrug-resistant Gram-negative bacterial infections in solid-organ transplant recipients recommends pre-transplant MDRO screening, empirical antimicrobial regimens guided by the recipient’s prior resistance profile, and structured post-transplant surveillance [15]. The organism-specific concordance observed in our cohort (Figure 1) provides direct empirical support for the pre-transplant screening and targeted prophylaxis components of this guidance, while the dominance of post-transplant acquisition, including among the 30% of recipients who were not colonized before transplantation, underscores the importance of the post-transplant infection prevention and stewardship measures the guideline emphasizes. The strong post-transplant mortality association we observed, even after time-dependent modeling, suggests that this post-transplant window is where intervention offers the greatest potential benefit: immunosuppression facilitates the transition from colonization to invasive disease [27,28,29,30] and prolonged mechanical ventilation, hemodialysis, and extended ICU stays create an environment conducive to MDRO acquisition [31,32,33,34,35]. That one-third of recipients without pre-transplant carriage acquired a resistant organism within the first year (22/66, Table 1) further underscores the need for targeted post-transplant interventions.

This study has several limitations. Its retrospective single-center design and relatively small sample size, including only 16 deaths, limit statistical power and generalizability. The low number of mortality events means that the absence of an association between pre-transplant ESBL/MDRO colonization and mortality should not be over-interpreted, and the multivariate estimates should be regarded with corresponding caution.

Residual confounding is possible because patients with post-transplant MDRO acquisition had more severe clinical courses characterized by prolonged ICU stay, mechanical ventilation, dialysis, and septic shock, and the exposure may be a mediator rather than an independent cause of mortality. Patients with the most severe pre-transplant MDRO burden may have died or been delisted before transplantation, so the pre-transplant findings are conditional on surviving to transplantation. Although the primary analysis modeled post-transplant MDRO exposure as a time-dependent covariate and the penalized model addressed complete separation, the resulting estimates depend on the penalty chosen and should be interpreted as confirming a robust association rather than a precise effect size. Misclassification of colonization or infection remains possible because surveillance cultures were performed according to institutional clinical practice rather than a fixed prospective protocol, and the 12-month exposure window, while accompanied by a 3-month sensitivity analysis, is longer than that used in some prior studies. Finally, mediation analysis distinguishing the independent and marker pathways was not feasible with the available events; prospective multi-center studies with prespecified time-dependent analyses are required to clarify the causal relationship between post-transplant MDRO acquisition and mortality.

## 5. Conclusions

In conclusion, in this contemporary single-center cohort of LT recipients, pre-transplant ESBL/MDRO colonization or infection was common and strongly predicted subsequent post-transplant MDRO acquisition, including infection with the same resistance phenotype, but was not associated with one-year mortality in this cohort, a finding that the limited number of deaths and the conditional nature of a transplant cohort preclude interpreting as a true null. Post-transplant MDRO status, and particularly invasive MDRO infection, was strongly and temporally associated with one-year mortality in time-dependent, landmark and 3-month-window analyses, though causality and independence from the complicated postoperative course remain to be established. These findings support the importance of monitoring MDRO dynamics throughout the transplant course: pre-transplant status should guide targeted prophylaxis and surveillance, while the post-transplant period represents the critical window for infection prevention and antimicrobial stewardship interventions. Prospective multi-center studies with prespecified time-dependent analyses and larger numbers of events are needed to clarify the causal relationship between post-transplant MDRO acquisition and mortality.

## Figures and Tables

**Table 1:** Baseline characteristics, clinical course, and outcomes of liver transplant recipients stratified by pre-transplant ESBL and MDRO colonization or infection.

*Characteristic*		Pre-Transplant ESBL/MDRO Colonization or Infection ^a^		*p*-Value
		Yes (*n* = 44)	No (*n* = 66)	
***Demographics and comorbidities***				
Age, mean ± SD		55 ± 12	54 ± 16	0.63
Gender (male)		27 (61%)	42 (64%)	0.84
Liver disease	NAFLD	15 (34%)	31 (47%)	0.24
	Alcoholic	4 (9%)	4 (6%)	0.71
	Viral hepatitis	7 (16%)	14 (21%)	0.62
	Autoimmune hepatitis	5 (11%)	9 (14%)	0.78
	Biliary disease	8 (18%)	8 (12%)	0.42
	Vascular	0	2 (3%)	0.52
	Metabolic	0	3 (4.5%)	0.27
	Other	8 (18%)	4 (6%)	0.06
Hepatocellular carcinoma		4 (9%)	17 (26%)	**0.045**
MELD		28 ± 7	25 ± 6	**0.004**
Diabetes mellitus		18 (41%)	25 (38%)	0.84
Hemodialysis		13 (29%)	1 (1.5%)	**<0.001**
Immune suppression prior to transplantation		15 (34%)	10 (15%)	**0.03**
Number of admissions in 2 years before transplantation		5.5 ± 4.5	2.8 ± 2.3	**<0.001**
ICU stay prior to transplantation		10 (23%)	4 (6%)	**0.02**
IV antibiotics 3 months prior to transplantation	None	15 (34%)	50 (76%)	**<0.001**
	Carbapenems	9 (21%)	1 (1.5%)	
	Other antibiotics	20 (45%)	15 (22.7%)	
ESBL infection 1Y prior to transplantation		18 (41%)	0	**<0.001**
MDRO colonization 1Y prior to transplantation	None	10 (23%)	66 (100%)	**<0.001**
	CRE	8 (18%)	0	
	CPE	9 (20.5%)	0	
	VRE	17 (37%)	0	
	MRSA	0	0	
Fungal colonization/infection 1 year prior to transplantation		9 (20.5%)	1 (1%)	**0.001**
***Transplantation course***				
Antibiotic prophylaxis protocol	Cefriaxone/Cefuroxime	4 (9%)	21 (32%)	**<0.001**
	Piperacillin + Tazobactam	29 (66%)	42 (64%)	
	Carbapenem ± aminoglycosides	11 (25%)	3 (4%)	
Duration pf prophylactic antibiotics		3.4 ± 1.7	2.9 ± 1.8	0.12
Blood transfusion during transplantation		7.3 ± 7.3	4.5 ± 8	**0.03**
Surgical complications	None	25 (57%)	41 (62%)	0.19
	Biliary	7 (21%)	8 (12%)	
	Vascular	2 (4%)	10 (15%)	
	Bleeding	3 (7%)	5 (8%)	
	Dehiscence	1 (2%)	1 (1.5%)	
	Late abdominal closure	1 (2%)	1 (1.5%)	
	Other	3 (7%)	0	
ICU LOS post-transplantation		17± 22	6 ± 7	**0.003**
Septic shock ^b^		14 (32%)	4 (6%)	**<0.001**
Hemodialysis		17 (39%)	9 (14%)	**0.005**
Mechanical ventilation > 72 h		18 (41%)	15 (23%)	**0.06**
Infectious complications post-transplantation		27 (62%)	19 (29%)	**<0.001**
Bloodstream infection		17 (39%)	4 (6%)	**<0.001**
Surgical site infection		5 (11.5%)	6 (9%)	1
Intrabdominal/bile infection		12 (27%)	9 (14%)	0.09
Other infections		14 (32%)	7 (11%)	**0.007**
MDRO or ESBL infection		22 (50%)	13 (20%)	**0.002**
Time from transplantation to infection (d, mean ± SD)		*n* = 22, 9.4 ± 6.2	*n* = 24, 13.5 ± 10	0.13
Time from transplantation to ESBL/MDRO infection (d, mean ± SD)		*n* = 21, 19 ± 30	*n* = 13, 12 ± 10	0.35
***1 year post-transplantation course***				
Number of hospital admissions		2.5 ± 3.0	2.7 ± 2.6	0.78
MDRO colonization	None	17 (39%)	44 (67%)	**0.015**
	CRE	3 (8%)	5 (7.5%)	
	CPE	14 (32%)	6 (9%)	
	VRE	10 (23%)	10 (15%)	
	MRSA	0	1 (1.5%)	
Number of infections		0.8 ± 1.3	0.75 ± 1.3	0.9
ESBL infection post-transplantation		19 (43%)	13 (20%)	**0.01**
MDRO infection post-transplantation	None	28 (64%)	63 (95%)	**<0.001**
	CRE	3 (7%)	1 (1.5%)	
	CPE	7 (16%)	2 (3%)	
	VRE	6 (14%)	0	
	MRSA	0	0	
Fungal infection post-transplantation		6 (14%)	3 (4.5%)	0.15
Adverse graft outcomes ^c^		19 (43%)	19 (29%)	0.15
One-year mortality		9 (21%)	7 (11%)	0.18

Bold values indicate statistical significance. Italics denote subheadings within the table. ESBL, extended-spectrum beta-lactamase; MDRO, multidrug-resistant organism; NAFLD, non-alcoholic fatty liver disease; MELD, Model for End-Stage Liver Disease; ICU, intensive care unit; CRE, carbapenem-resistant *Enterobacterales*; CPE, carbapenemase-producing *Enterobacterales*; VRE, vancomycin-resistant *Enterococcus*; MRSA, methicillin-resistant *Staphylococcus aureus*; LOS, length of stay. ^a^ Pre-transplant MDRO status (colonization and/or infection) and pre-transplant ESBL infection within 12 months prior to transplantation were grouped together as pre-transplant ESBL/MDRO colonization or infection. ^b^ Septic shock was defined as infection-related organ dysfunction that despite adequate fluid resuscitation required vasopressors to maintain a mean arterial pressure ≥ 65 mmHg and a lactate > 2 mmol/L. ^c^ Primary failure, rejection, or re-transplantation during the index hospitalization or within 12 months following transplantation.

**Table 2:** Baseline characteristics, clinical course, and outcomes of liver transplant recipients stratified by post-transplant MDRO colonization or infection.

Characteristic		Post-Transplant MDRO Status ^a^		*p*-Value
		Yes (*n* = 49)	No (*n* = 61)	
***Demographics and comorbidities***				
Age, mean ± SD		58 ± 13	51 ± 16	**0.006**
Gender (male)		34 (70%)	35 (57%)	0.24
Liver disease	NAFLD	25 (51%)	20 (33%)	0.08
	Alcoholic	5 (10%)	3 (5%)	0.46
	Viral hepatitis	8 (16%)	13 (21%)	0.63
	Autoimmune hepatitis	5 (10%)	9 (15%)	0.57
	Biliary disease	5 (10%)	11 (18%)	0.29
	Vascular	1 (2%)	1 (1.5%)	1
	Metabolic	1 (2%)	3 (5%)	0.63
	Other	6 (12%)	6 (10%)	0.76
Hepatocellular carcinoma		7 (14%)	14 (23%)	0.33
MELD		27 ± 6	26 ± 6	0.26
Diabetes mellitus		25 (51%)	18 (30%)	0.03
Hemodialysis		13 (26%)	1 (1.5%)	**<0.001**
Immune suppression prior to transplantation		11 (22%)	14 (23%)	1
Number of total transplantations		1.14 ± 0.46	1.1 ± 0.4	0.58
Number of admissions in 2 years before transplantation		4.5 ± 4.0	3.5 ± 3.1	0.16
ICU stay prior to transplantation		8 (16%)	6 (10%)	0.39
IV antibiotics 3 months prior to transplantation	None	22 (45%)	43 (70%)	**0.009**
	Carbapenems	8 (16%)	2 (3%)	
	Other antibiotics	19 (37%)	16 (26%)	
ESBL infection 1Y prior to transplantation		15 (30%)	11 (18%)	0.17
MDRO colonization 1Y prior to transplantation	None	23 (47%)	53 (86%)	**0.0003**
	CRE	7 (14%)	1 (2%)	
	CPE	8 (16%)	1 (2%)	
	VRE	11 (22%)	6 (10%)	
	MRSA	0	0	
Fungal colonization/infection 1Y pre-transplantation		7 (14%)	3 (5%)	0.11
***Transplantation course***				
Hospital LOS prior to transplantation		12 ± 22	4 ± 6	**0.025**
ICU LOS prior to transplantation		6 ± 14	0.5 ± 2	**0.003**
Antibiotics prophylaxis protocol	Cefriaxone/Cefuroxime	10 (20%)	15 (24%)	0.02
	Piperacillin–Tazobactam	28 (57%)	43 (70%)	
	Carbapenems/aminoglycosides	11 (22%)	3 (5%)	
Duration of prophylactic antibiotics		3.4 ± 1.8	3.0 ± 1.7	0.26
Blood transfusions during surgery (mean ± SD)		7 ± 7	5 ± 6	**0.055**
Surgical complications	None	23 (47%)	43 (70%)	**0.042**
	Biliary	11 (22%)	6 (10%)	
	Vascular	5 (10%)	7 (11.5%)	
	Bleeding	6 (12%)	2 (3%)	
	Dehiscence	2 (4%)	0	
	Late abdominal closure	0	2 (3%)	
	Other	2 (4%)	1 (1.5%)	
ICU LOS		18 ± 22	5 ± 5	**<0.001**
Septic shock ^b^		15 (30%)	3 (5%)	**<0.001**
Hemodialysis		20 (40%)	6 (10%)	**<0.001**
Mechanical ventilation > 72 h		23 (47%)	10 (16%)	**<0.001**
Infectious complications		34 (70%)	12 (20%)	**<0.001**
Bloodstream infection		17 (34%)	4 (6.5%)	**<0.001**
Surgical site infection		9 (18%)	2 (3%)	**0.01**
Intrabdominal/bile infections		18 (36%)	3 (5%)	**<0.001**
Other infections		16 (32%)	5 (8%)	**0.001**
MDRO or ESBL infection		28 (57%)	7 (12%)	**<0.001**
Time from transplantation to infection (mean ± SD)		*n* = 34, 10.5 ± 8	*n* = 12, 12.8 ± 9.3	0.4
Time from transplantation to ESBL/MDRO infection (d, mean ± SD)		*n* = 27, 18.4 ± 27	*n* = 7, 8.6 ± 6	0.36
***1 year post-transplantation course***				
Number of hospital admissions (d, mean ± SD)		3.5 ± 3.2	2.0 ± 2.2	**0.007**
MDRO colonization	None	0	61 (100%)	**<0.001**
	CRE	8 (16%)	0	
	CPE	20 (41%)	0	
	VRE	20 (41%)	0	
	MRSA	1 (1%)	1 (2%)	
Infection (any) mean ± SD		1.2 ± 1.6	0.4 ± 0.94	**0.004**
ESBL infection		25 (31%)	7 (11.5%)	**<0.001**
MDRO infection	None	30 (62.5%)	61 (100%)	**<0.001**
	CRE	4 (8%)	0	
	CPE	9 (18%)	0	
	VRE	6 (12%)	0	
	MRSA	0		0
Fungal infection post-transplantation		8 (16%)	1 1.5%)	**0.01**
Adverse graft outcomes ^c^		18 (37%)	20 (33%)	0.7
Need for hemodialysis > 30 d		15 (30%)	4 (6.5%)	**0.002**
One-year mortality		14 (29%)	2 (3%)	**<0.001**

Bold values indicate statistical significance. Italics denote subheadings within the table. MDRO, multidrug-resistant organism; NAFLD, non-alcoholic fatty liver disease; MELD, Model for End-Stage Liver Disease; ICU, intensive care unit; ESBL, extended-spectrum beta-lactamase; CRE, carbapenem-resistant *Enterobacterales*; CPE, carbapenemase-producing *Enterobacterales*; VRE, vancomycin-resistant *Enterococcus*; MRSA, methicillin-resistant *Staphylococcus aureus*; LOS, length of stay. ^a^ Post-transplant MDRO status included any new colonization or infection identified during the index hospitalization or within 12 months following transplantation. ^b^ Septic shock was defined as infection-related organ dysfunction that despite adequate fluid resuscitation required vasopressors to maintain a mean arterial pressure ≥ 65 mmHg and a lactate >2 mmol/L. ^c^ Primary failure, rejection, or re-transplantation during the index hospitalization or within 12 months following transplantation.

## Data Availability

The original contributions presented in this study are included in the article. Further inquiries can be directed to the corresponding author.

## Supplementary Materials

The following supporting information can be downloaded at https://www.mdpi.com/article/10.3390/diseases14100362/s1, Table S1: Operational definitions of multidrug-resistant organisms and ESBL, Table S2: Infection sites of MDRO/ESBL infections before and after liver transplantation, Table S3: Timing of post-transplant MDRO/ESBL colonization/infection and subsequent mortality among liver transplant recipients who died during follow-up (*n* = 16), Table S4: Time-dependent Cox proportional hazards models for one-year mortality, Figure S1: One-year survival by post-transplant MDRO status.
