# Supplementary material for: The Impact of Pre- Versus Post-Transplant Multidrug-Resistant Organism Colonization on Liver Transplant Outcomes: A Retrospective Cohort Study
Source: Diseases. 2026 Oct 1;14(10):362. doi: 10.3390/diseases14100362 (PMC13702940; doi:10.3390/diseases14100362)
Supplement: Supplementary file 1 [file diseases-14-00362-s001.zip › diseases-4537585-supplementary.pdf]

**The impact of pre- versus post-transplant multidrug-resistant organism colonization on liver transplant outcomes: A retrospective cohort study**

Emil Aliev, Efrat Orenbuch-Harroch, Abed Khalaileh, Ashraf Imam, Matan Joel Cohen, Maya Korem

**Supplementary Table S1.** Operational definitions of multidrug-resistant organisms and ESBL

| **Organism group** | **Operational definition** | **Detection method** |
|---|---|---|
| MRSA | Oxacillin non-susceptibility or presence of the mecA determinant | Automated susceptibility testing (CLSI) |
| VRE | Vancomycin MIC ≥32 mg/L or presence of the vanA/vanB determinant | Automated susceptibility testing (CLSI) |
| CRE | Non-susceptibility to at least one carbapenem, without identification of a carbapenemase | Automated susceptibility testing (CLSI) |
| CPE | Carbapenemase gene detected | Multiplex PCR |
| ESBL (phenotypic) | Reduced susceptibility or resistance to third-generation cephalosporins (ceftriaxone or ceftazidime) | Automated susceptibility testing (CLSI) |

Definitions applied to all isolates; susceptibility testing per CLSI guidelines (M100, 35th ed.); carbapenemase genes detected by multiplex PCR.

MRSA, methicillin-resistant Staphylococcus aureus; VRE, vancomycin-resistant Enterococcus; CRE, carbapenem-resistant Enterobacterales; CPE, carbapenemase-producing Enterobacterales; ESBL, extended-spectrum β-lactamase; MIC, minimum inhibitory concentration; PCR, polymerase chain reaction.

**Supplementary Table S2.** Infection sites of MDRO/ESBL infections before and after liver transplantation[a]

| **Period** | **Site** | **n** | **% of patients with cultures/infections** |
|---|---|---|---|
| Pre-transplant (n=46 with positive clinical cultures)[b,c] | Urine / respiratory | 34 | 74% |
| | Bloodstream | 17 | 37% |
| | Peritoneal fluid / bile | 15 | 33% |
| | Wound | 2 | 4% |
| Post-transplant (n=48 with positive clinical culture)[d,e] | Intra-abdominal / biliary | 20 | 42% |
| | Bloodstream | 19 | 40% |
| | Urinary / respiratory | 17 | 35% |
| | Surgical site | 9 | 19% |

MDRO; multidrug resistant organism, ESBL; extended-spectrum beta-lactamase producing bacteria.

[a]Patients may appear in more than one site category; percentages therefore do not sum to 100.
[b]In 12 months before transplantation
[c]Pre-transplant clinical cultures were documented in 46 patients; pre-transplant ESBL infection was documented in 26 patients
[d]In 12 months after transplantation

[e]Among the 48 patients with a post-transplant MDRO/ESBL infection, the causative MDRO organisms were CPE (n=9), VRE (n=6) and CRE (n=4); an additional 32 patients had a post-transplant ESBL infection (some patients had both ESBL and MDRO infections)

**Supplemental Table S3.** Timing of post-transplant MDRO/ESBL colonization/infection and subsequent mortality among liver transplant recipients who died during follow-up (n=16)

| Mortality cases (n=16) | Days from LT to death | Infection-related deaths | First post LT MDRO/ESBL infection (days) | First post LT MDRO colonization (days) |
|---|---|---|---|---|
| 1 | 49 | Yes | 21 | Pre-transplant |
| 2 | 316 | Yes | 4 | Pre-transplant |
| 3 | 38 | Yes | 16 | None |
| 4 | 65 | Yes | 18 | 31 |
| 5 | 41 | Yes | 7 | Pre-transplant |
| 6 | 227 | Yes | 51 | Pre-transplant |
| 7 | 56 | Yes | 9 | 45 |
| 8 | 71 | Yes | None | None |
| 9 | 49 | Yes | 5 | 7 |
| 10 | 347 | Yes | None | 346 |
| 11 | 42 | No | 20 | Pre-transplant |
| 12 | 81 | Yes | None | 31 |
| 13 | 85 | Yes | 28 | 29 |
| 14 | 4 | No | None | None |
| 15 | 264 | Yes | None | 248 |
| 16 | 191 | No | 3 | Pre-transplant |

LT; liver transplantation, MDRO; multidrug resistant organism, ESBL; extended-spectrum beta-lactamase producing bacteria.

**Supplementary Table S4** Time-dependent Cox proportional hazards models for one-year mortality[a]

| **Model** | **Covariate** | **HR** | **95% CI** | **p** |
|---|---|---|---|---|
| 12-month window, univariable (penalized) | Post-transplant MDRO status | 4.32 | 1.93–9.63 | <0.001 |
| 12-month window, adjusted | Post-transplant MDRO status | 4 | 1.77–9.03 | <0.001 |
| | Age (per year) | 1.06 | 0.99–1.12 | 0.09 |
| | MELD score (per point) | 1.01 | 0.91–1.12 | 0.85 |
| 30-day landmark analysis (n=105) | Post-transplant MDRO (cultures before day 30) | 3.15 | 1.08-9.19 | 0.035 |
| 3-month window, univariable (penalized) | Post-transplant MDRO status | 4.04 | 1.77–9.21 | 0.001 |
| 3-month window, adjusted (penalized) | Post-transplant MDRO status | 3.73 | 1.62–8.59 | 0.002 |
| | Age (per year) | 1.06 | 0.99–1.15 | 0.1 |
| | MELD score (per point) | 1.11 | 0.96–1.27 | 0.17 |

HR, hazard ratio; CI, confidence interval; MDRO; multidrug resistant organism, MELD, Model for End-Stage Liver Disease.

[a] Post-transplant MDRO exposure entered as a time-dependent covariate beginning at the first positive MDRO culture; follow-up censored at 365 days. Penalized (Firth-type) models were used where complete separation occurred (univariable models); the adjusted 12-month model converged without penalization. Landmark analysis: patients alive at day 30, exposure defined by cultures before day 30. Because 14 of the 16 deaths occurred after MDRO acquisition, the univariable 12-month and 3-month models exhibited complete separation and were fitted with a Firth-type penalty; the adjusted 12-month model is unpenalized. In the 12-month adjusted model, age retained significance and MELD did not; in the penalized 3-month models neither covariate reached significance, consistent with reduced power in the restricted window.

**Supplementary Figure S1.** One-year survival by post-transplant MDRO status

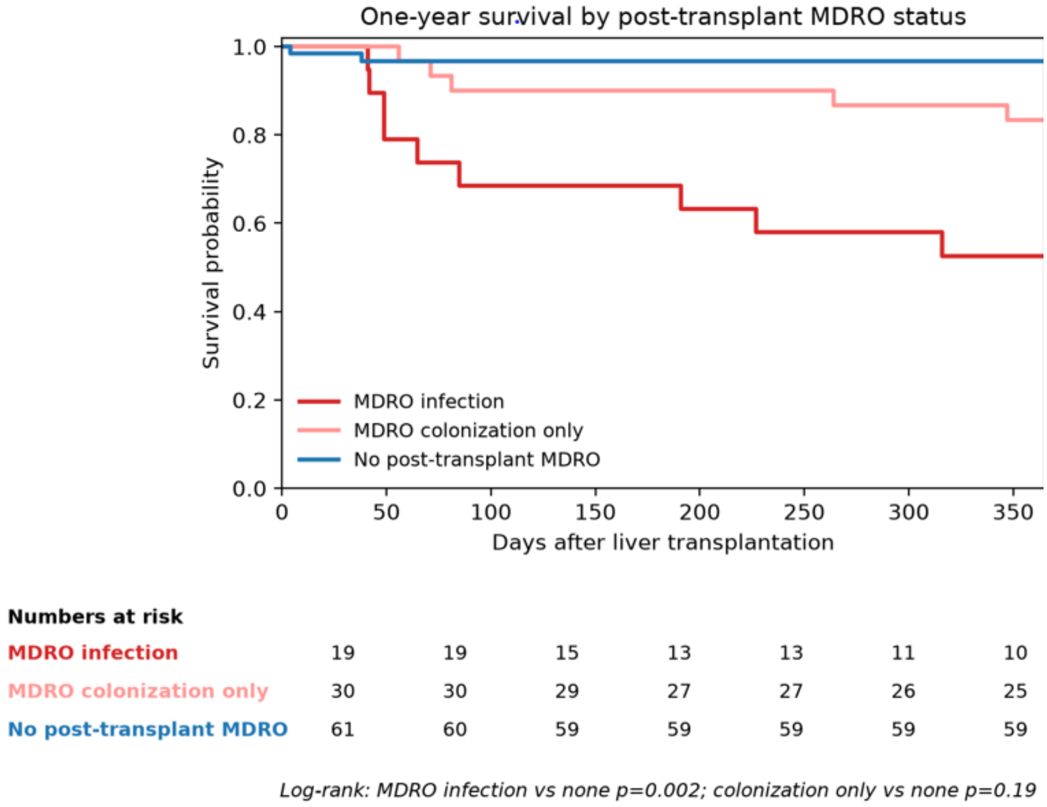


One-year post-transplant survival stratified by post-transplant MDRO infection (n=19, 9 deaths), MDRO colonization alone (n=30, 5 deaths), and no post-transplant MDRO (n=61, 2 deaths), with numbers at risk (log-rank: MDRO infection vs none p=0.002; colonization alone vs none p=0.19).
